# Low vision services in sub-Saharan Africa: a systematic review

**DOI:** 10.3389/frhs.2026.1839924

**Published:** 2026-06-03

**Authors:** Kwadwo Owusu Akuffo, Sylvester Kyeremeh, Sylvia Agyekum, Isaiah Osei Duah Junior, Josephine Ampong, Mallissa Okine, Albert Kwadjo Amoah Andoh, Gabriel Kwaku Agbeshie, Josephine Ampomah Boateng, Nana Akwasi Owusu Mensah, Werner Eisenbarth

**Affiliations:** 1 Department of Optometry and Visual Science, College of Science, Kwame Nkrumah University of Science and Technology, Kumasi, Ghana; 2 Department of Psychology, John R. and Kathy R. Hairston College of Health and Human Sciences, North Carolina Agricultural and Technical State University, Greensboro, NC, United States; 3 Department of Applied Science and Mechatronics, HM Hochschule München University of Applied Sciences, Munich, Germany

**Keywords:** barriers, facilitators, magnifiers, rehabilitation, residual vision, telescopes, vision aids

## Abstract

**Background:**

Understanding the availability, access, and outcomes of low vision services (LVS) in Sub-Saharan Africa (SSA) is essential to reduce the burden and enhance the quality of life for patients with residual vision. The aim of the study is to systematically evaluate the availability, accessibility, utilization, and functional outcomes of LVS in SSA from both provider and patient perspectives.

**Methods:**

A comprehensive systematic search was conducted across five databases—PubMed, Scopus, Web of Science, African Journals Online, and Google Scholar—from inception through June 2025, following PRISMA guidelines. Studies reporting on any aspect of LVS delivery within SSA were eligible for inclusion. Extracted data were synthesized narratively, and methodological quality was rigorously assessed using the Joanna Briggs Institute Critical Appraisal Checklists.

**Results:**

Eleven studies from six SSA countries met the inclusion criteria, with an equal number focusing on service providers and patients (including one overlapping study). Overall, LVS were found to be limited and unevenly distributed across the region, with Ghana demonstrating the most comprehensive coverage. While basic clinical assessments were commonly reported, access to advanced diagnostic evaluations and comprehensive rehabilitation services remained scarce. Optical aids such as magnifiers and telescopes were more widely available than non-optical devices or structured training programs. Studies assessing functional outcomes reported significant improvements in visual acuity, particularly for distance vision, following intervention. However, multiple barriers to service delivery were identified, including limited infrastructure, insufficient provider training, high costs of devices, financial constraints, low public awareness, and persistent social stigma surrounding vision impairment.

**Conclusion:**

The LVS in SSA are still very limited, even though studies report measurable improvements in visual acuity following intervention. There is an urgent need to invest in training health workers, integrating services, making devices affordable, and increasing public awareness. Improving these services is crucial for fair access to eye care and achieving Universal Health Coverage in the region.

**Systematic Review Registration:**

https://www.crd.york.ac.uk/PROSPERO/view/CRD420251112575, PROSPERO CRD420251112575.

## 1 Introduction

Over 339 million people experience visual impairment, including 295 million with low vision and 43.3 million who are blind worldwide (1). Although SSA accounts for only 14.1% of the world's population, it bears nearly 20% of this residual vision burden (2). Patients with low vision have a visual acuity ≤6/18 or a visual field of less than 20 degrees in the better eye, that cannot be corrected by conventional spectacles, contact lenses, or medical intervention (3, 4), that significantly impair functional capacity, quality of life, and reduce education, employment, and social engagements (3).

Low vision services (LVS) are designed to optimize residual vision and enhance individual autonomy through comprehensive clinical assessments, the provision of optical and non-optical assistive devices, and structured rehabilitation programs that include psychosocial support (5, 6). Despite their well-documented benefits, global access to LVS remains critically low, with only 5%–10% of individuals who require these services actually receiving them (7). The LVS gap is largely attributed to a range of barriers, including limited public awareness, a shortage of trained professionals, underdeveloped service infrastructure, fragmented referral networks, and sociocultural factors such as stigma and skepticism about the effectiveness of LV interventions (7–9).

In SSA where the burden of visual impairment is particularly pronounced, these challenges are further compounded by regional disparities in healthcare access and resource allocation (10). The alarmingly high prevalence of low vision in SSA, alongside persistently low rates of LVS utilization, constitutes a significant public health issue that necessitates urgent and comprehensive examination (2, 11). Many individuals across the region continue to experience limited access to essential rehabilitative care due to systemic challenges such as inadequate service coverage, financial hardship, geographic isolation, and insufficient human resources (12, 13).

Herein, the present systematic review was conducted to address three key objectives: (1) to assess the availability and geographic distribution of LVS across SSA; (2) to evaluate access-related factors and utilization patterns among individuals with low vision; and (3) to examine reported functional outcomes and the characteristics of existing service delivery models. By synthesizing evidence from multiple national and subnational contexts, this review aims to provide a robust evidence base to inform policy development, improve clinical and service delivery practices, and identify priority areas for future research to enhance the quality, accessibility, and equity of low vision care in the region.

## 2 Methods

**Figure 1:**
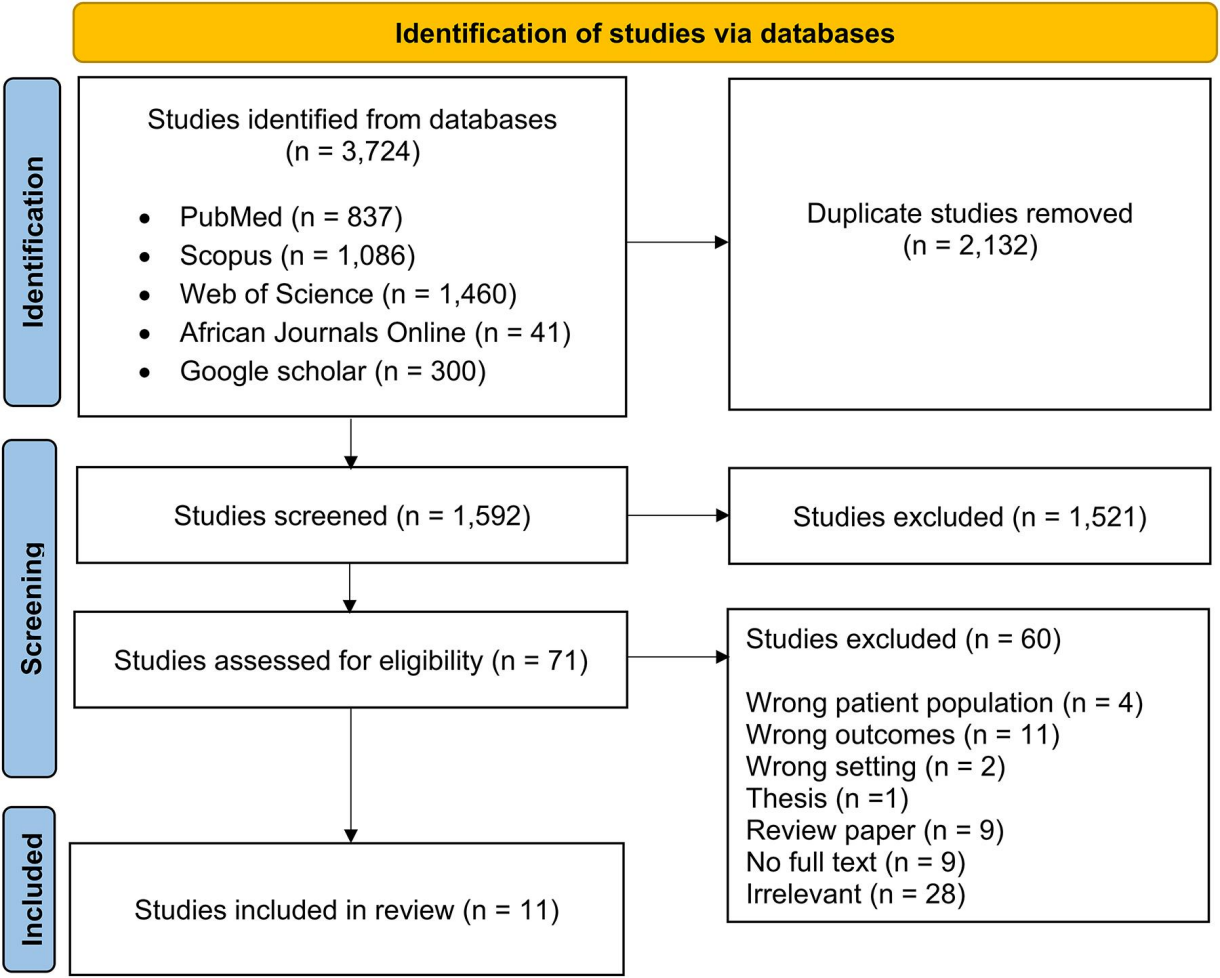
PRISMA flow diagram.

The review followed the Preferred Reporting Items for Systematic Reviews and Meta-Analyses (PRISMA) (14) reporting guidelines to ensure transparency in reporting and was prospectively registered in the PROSPERO (CRD420251112575). The selection of studies is delineated by the PRISMA flow diagram (see Figure 1).

### 2.1 Eligibility criteria

Primary studies reporting on the availability, access, utilization, or outcomes of low vision services in SSA were included. Eligible studies involved individuals with low vision or service providers and reported on at least one of the following: types of services, geographic distribution, access barriers or facilitators, or functional outcomes. All study designs including quantitative, qualitative, and mixed methods were considered. Studies were excluded if they focused solely on blindness or general eye care without specific reference to low vision services, or if they were reviews, editorials, or conducted outside the SSA region.

### 2.2 Information sources

A comprehensive search across five electronic databases: PubMed, Web of Science, Scopus, African Journals Online, and Google Scholar were conducted. Systematic searches were performed from inception through June 2025 without language restrictions. Search terms were structured around three concepts, which includes “Sub-Saharan Africa,” “low vision,” and “low vision services.” Boolean operators (AND, OR) were used to combine terms across domains. The full search strategy including all database-specific syntax, is provided in Supplementary File S1.

### 2.3 Study selection and data extraction

Titles, abstracts, and full texts were independently screened for eligibility by two reviewers using Covidence software. Studies were selected based on relevance to the availability, access, utilization, or outcomes of LVS in SSA. Data were extracted into a standardized form and organized under provider-reported and patient-reported themes. Extracted variables for both study types included study characteristics (author, year, country, setting, study design, sample size or population demographics), and outcomes related to low vision service provision and utilization.

For provider-based studies, data included availability and distribution of services, types of clinical assessments performed, availability of equipment and assistive devices, provision of rehabilitation services, and reported barriers and facilitators to service provision and uptake. Data were collected using structured or semi-structured questionnaires administered at a single time point. Administration methods varied across studies and included self-administered (online or paper-based) and interviewer-assisted approaches, with distribution occurring via electronic platforms, in-person delivery, or professional meetings.

For patient-based studies, data included causes and severity of visual impairment, types and extent of services accessed, access and utilization patterns, pre- and post-intervention visual outcomes, and patient-reported barriers and facilitators to service uptake. All data were extracted independently by two reviewers (JA and MO). Discrepancies were resolved through discussion and consensus, with a third reviewer (SA) consulted when necessary.

### 2.4 Data synthesis

Data were synthesized without meta-analysis and structured around the three primary research questions: (1) service availability and geographic distribution, (2) access and utilization, and (3) functional outcomes and effectiveness. Studies were first grouped according to provider perspective and patient perspective. Within each domain, findings were organized and compared across studies to identify patterns, consistencies, and variations in reported outcomes and service characteristics.

**Figure 2:**
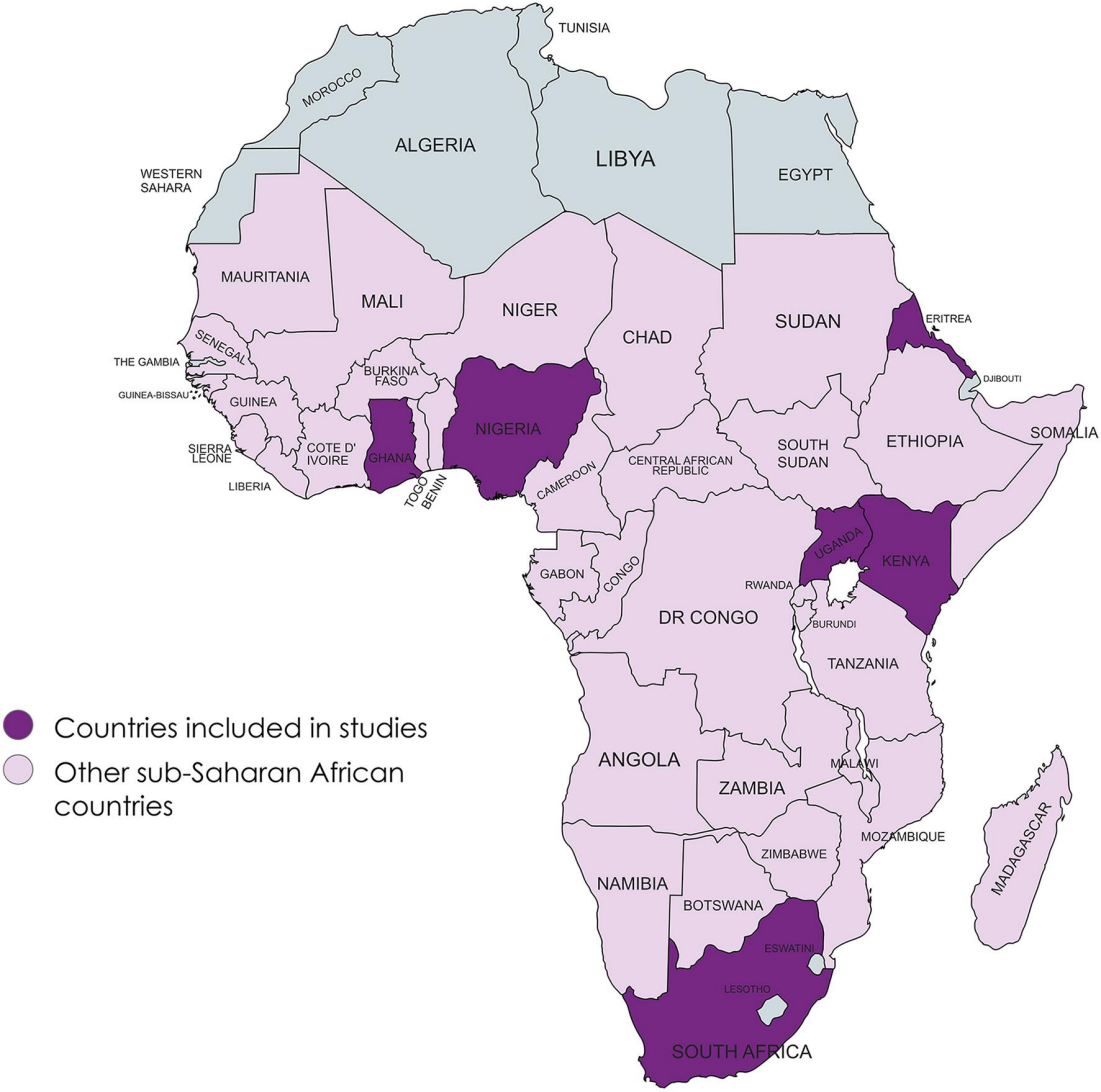
Distribution of included studies in the sub-Saharan Africa.

Quantitative findings were summarized using descriptive statistics such as frequencies and percentages. Comparative tables were constructed to highlight patterns across studies and regions. For the assessment of low vision service components (Table 3), facility-level data reported across studies were aggregated using a simple summation approach. Specifically, the number of facilities reporting each service component was summed across studies, and percentages were calculated using the total number of facilities as the denominator. Barriers and facilitators were analyzed thematically to identify recurrent patterns. Where applicable, changes in visual acuity from pre- to post-intervention were illustrated using slope charts. A choropleth map of sub-Saharan Africa was developed to visually represent the regional distribution and availability of LVS as shown in Figure 2.

### 2.5 Quality assessment

The methodological quality and risk of bias of all included studies were assessed using the Joanna Briggs Institute (JBI) Critical Appraisal Checklists. Specifically, the JBI Checklist for Analytical Cross-Sectional Studies (15) was applied across all studies. This tool evaluates key methodological aspects, including clarity of inclusion criteria, representativeness of the sample, validity and reliability of outcome measures, appropriateness of statistical analyses, and adequacy of response rates. Each study was assigned an overall risk of bias rating as “low,” “moderate,” or “high.” Quality assessments were conducted independently by two reviewers (JA and MO), with discrepancies resolved through discussion and consensus.

## 3 Results

### 3.1 Study selection

The 3,724 records were identified through database searches. Following the removal of duplicates, titles and abstracts were screened for relevance, resulting in 71 full-text articles assessed for eligibility. Ultimately, 11 studies met the inclusion criteria and were included in the review (see Figure 1). These studies examined LVS in SSA from both patient and provider perspectives.

### 3.2 Study characteristics

The characteristics of the 11 included studies are presented below (see Tables 1 and 2). One study (16) contributed data to both provider and patient perspectives and was counted in both categories Six studies focused on provider perspectives and were conducted in Ghana (*n* = 4) and Nigeria (*n* = 2). All employed cross-sectional survey designs and involved various eye care professionals, including optometrists, ophthalmic nurses, and ophthalmologists. Study settings included public hospitals, private practices, and mission-based or non-governmental clinics. Sample sizes ranged from 25 to 213 participants (See Table 1).

**Table 1:** Study characteristics of included studies from providers.

Study ID	Author (year)	Country	Study design	Setting	Participants	Sample size
1	Nyankerh et al. (2019) (17)	Ghana	Cross-sectional	Private practice = 101 (78.9%)	Optometrists	128
				Government practice = 17 (13.3%)		
				Other = 10 (7.8%)		
2	Boadi-Kusi et al. (2015) (18)	Ghana	Cross-sectional	Public facilities = 51%	Optometrists	90
				Private facilities = 49%		
3	Okoye et al. (2007) (19)	Nigeria	Cross-sectional	Teaching hospital = 63 (75.9%)	Ophthalmologists (Fellows and residents)	83
				Non-teaching hospitals = 20 (24.1%)		
4	Kyeremeh et al. (2021) (20)	Ghana	Cross-sectional	Public facilities = 35 (64.8%)	Optometrists Ophthalmic nurses Ophthalmologists	54
				Private facilities = 16 (29.6%)		
				NGO = 3 (5.5%)		
5	Akuffo et al. (2025) (13)	Ghana	Cross-sectional	Private facility = 112 (52.6%)	Optometrists	213
				Public facility = 62 (29.1%)		
				NGO = 5 (2.3%)		
				CHAG = 34 (16%)		
6	Monye et al. (2020) (16)	Nigeria	Cross-sectional	9 tertiary hospitals	Optometrists Ophthalmic nurses Ophthalmologists	25

NGO, non-governmental organization; CHAG, Christian Health Association of Ghana.

The six patient-focused studies were conducted in South Africa, Ghana, Nigeria, Eritrea, Kenya, and Uganda. Three were facility-based (eye clinics or hospitals), while the remaining three were conducted in schools for the blind. Sample sizes ranged from 10 to 621 participants. Most studies included both male and female participants across a wide age range—from young children (as young as 4 years) to older adults (up to 100 years). Methodologies included retrospective, prospective, and cross-sectional designs as shown in Table 2.

**Table 2:** Study characteristics of included studies from patients.

Study ID	Author (year)	Country	Study design	Setting	Sample size	Age range
7	Mabunda et al. (2020) (21)	South Africa	Retrospective	University of KwaZulu-Natal low vision clinic	621	4–93 years
6	Monye et al. (2020) (16)	Nigeria	Cross-sectional	9 Tertiary Hospitals	10	<15 to >65 years
8	Ilechie et al. (2020) (22)	Ghana	Prospective cross-sectional	3 Schools for Blind	252	5–15 years
9	Gyawali et al. (2018) (23)	Eritrea	Cross-sectional	School for Blind	86	6–17 years
10	Eze et al. (2018) (24)	Nigeria	Cross-sectional	Eye clinic of University of Nigeria Teaching Hospital	197	5–100 years
11	Silver et al. (1995) (25)	Kenya, Uganda	Not reported	Schools for Blind	216	5–22 years

### 3.3 Availability and distribution of low vision services

Table 3 present findings from the six provider-based studies reporting on the availability and distribution of low vision services. Across these studies, service availability was low and unevenly distributed geographically. The number of facilities (73 in total) providing low vision services were reported in 4 studies, 3 from Ghana (13, 17, 20) and 1 from Nigeria (16). As the total reflects a simple summation of facilities across studies, the number may be overstated due to possible duplication, particularly among the Ghanaian studies. Clinical services commonly provided included visual acuity assessment (distance and near), refraction, verification of prescriptions, and occasionally visual field testing and contrast sensitivity measurement.

**Table 3:** Assessment components in low vision facilities reported in four studies.

Assessment category	*n* (%)
History and symptoms	
Visual history	20 (27.4%)
Ocular history	16 (21.9%)
Medical history	16 (21.9%)
Social history	14 (19.2%)
Duration of symptoms	14 (19.2%)
Other disabilities	13 (17.8%)
Visual symptoms	15 (20.5%)
Ocular symptoms	15 (20.5%)
Medical symptoms	14 (19.2%)
Social symptoms	14 (19.2%)
Needs and goal setting	
Distance tasks	13 (17.8%)
Near tasks	14 (19.2%)
Mobility	11 (15.1%)
Daily living skills	10 (13.7%)
Current assistive devices	11 (15.1%)
Support needs	9 (12.3%)
Treatment needs	9 (12.3%)
Other needs	7 (0.96%)
Clinical assessment	
Distance visual acuity	56 (76.7%)
Near/reading visual acuity	55 (75.3%)
Distance prescription verification	49 (70%)
Near prescription verification	48 (67.1%)
Retinoscopy	12 (16.4%)
Distance refraction	52 (71.2%)
Near refraction	50 (68.5%)
Accommodation	7 (0.96%)
Binocular vision	16 (21.9%)
Magnification establishment	9 (12.3%)
Contrast sensitivity	5 (0.68%)
Glare function	5 (0.68%)
Color vision	28 (38.4%)
Visual field testing	42 (57.5%)
LV assistive devices assessment	9 (12.3%)
LV devices dispensing	29 (39.7%)
Device use training	28 (38.4%)
Advice and referral	13 (17.8%)
Other assessments	5 (6.8%)

*n*, frequency; %, percent frequency; LV, low vision.

### 3.4 Low vision services

Table 4 provides an overview of LVS reported in eight of the included studies, categorized into optical aids, non-optical aids, and rehabilitation services. Optical aids were the most frequently documented, with magnifiers being the most commonly available, reported in 29.6%–100% of facilities across studies. Telescopes were less frequently available, ranging from 25.0% to 38.2%, while more advanced devices such as closed-circuit televisions (CCTVs) were available in only 25.0% of settings or limited to a small number of units. Non-optical aids including sunglasses, hats, and face caps showed moderate availability, reported in 24.5%–42.1% of the studies. However, several studies indicated a complete lack of these aids in some facilities. Rehabilitation services were the least consistently available. Counseling services were reported in a few studies, with availability ranging from 6.4% to 100%. Referrals were rarely documented (1.9%–3.7%), and orientation and mobility training was available in only 22%–57% of facilities, highlighting a significant gap in comprehensive low vision rehabilitation.

**Table 4:** Summary of low vision devices and rehabilitation services reported across included studies.

Study reference	Optical aids (%)	Non-optical aids (%)	Rehabilitation services (%)
Mabunda et al. (2020) (21)	Telescopes = 210 (33.8%) Magnifiers = 184 (29.6%)	Sunglasses/hats = 152 (24.5%)	Counselling/referral to blind school = 40 (6.4%). Referral to ophthalmologist = 23 (3.7%). Referral to OT = 12 (1.9%)
Monye et al. (2020) (16)	Electronic LVD = 1 (11%) Non-optical LVD = 2 (22%) Fillers = 2 (22%) Handheld monocular telescopes = 4 (44%) Dome and bar magnifiers = 4 (44%) Stand magnifiers = 4 (44%) Foldable and handheld magnifiers = 4 (44%) Spectacle magnifiers = 3 (33%)	None reported	LVD use counselling = 9 (100%). LVD training = 2 (22%) Vocational advice = 8 (89%) Environmental modification = 6 (67%) Orientation and mobility training = 2 (22%)
Ilechie et al. (2020) (22)	Spectacles = 2 Stand magnifier = 6 Hand-held magnifier = 22 Tele-microscopes = 4 Spectacle magnifiers = 3	None reported	None reported
Gyawali et al. (2018) (23)	Spectacles = 29 (33.7%) Spectacle magnifiers = 9 (10.5%) Stand magnifier = 6 (7.0%) Hand-held magnifier = 3 (3.5%) Telescopes = 24 (27.9%)	None reported	None reported
Eze et al. (2018) (24)	Telescopes = 44 (38.2%) Magnifiers = 17 (14.5%)	Face caps/antiglare glasses/table lamps = 83 (42.1%)	None reported
Silver et al. (1995) (25)	Stand magnifiers = 21 (42%) High reading adds = 19 (38%) Hand-held magnifiers = 10 (20%)	None reported	Braille training = 80 (57%)
Nyankerh et al. (2019) (17)	Handheld magnifiers = 2 (50%) Stand magnifiers = 1 (25%) Telescopes = 1 (25%) Magnifying lenses = 4 (100%) CCTV = 1 (25%) Others = 2 (50%)	None reported	None reported
Kyeremeh et al. (2021) (20)^a^	Spectacle magnifiers = 4 (23.5%) Foldable/hand-held magnifiers = 8 (47.1%) Stand magnifiers = 4 Dome/bar magnifiers = 3 Hand-held telescopes = 3 CCTV = 2	None reported	None reported

a Number of facilities with LVD, LVD, low vision devices; CCTV, closed-circuit television.

### 3.5 Functional outcomes of low vision services

**Figure 3:**
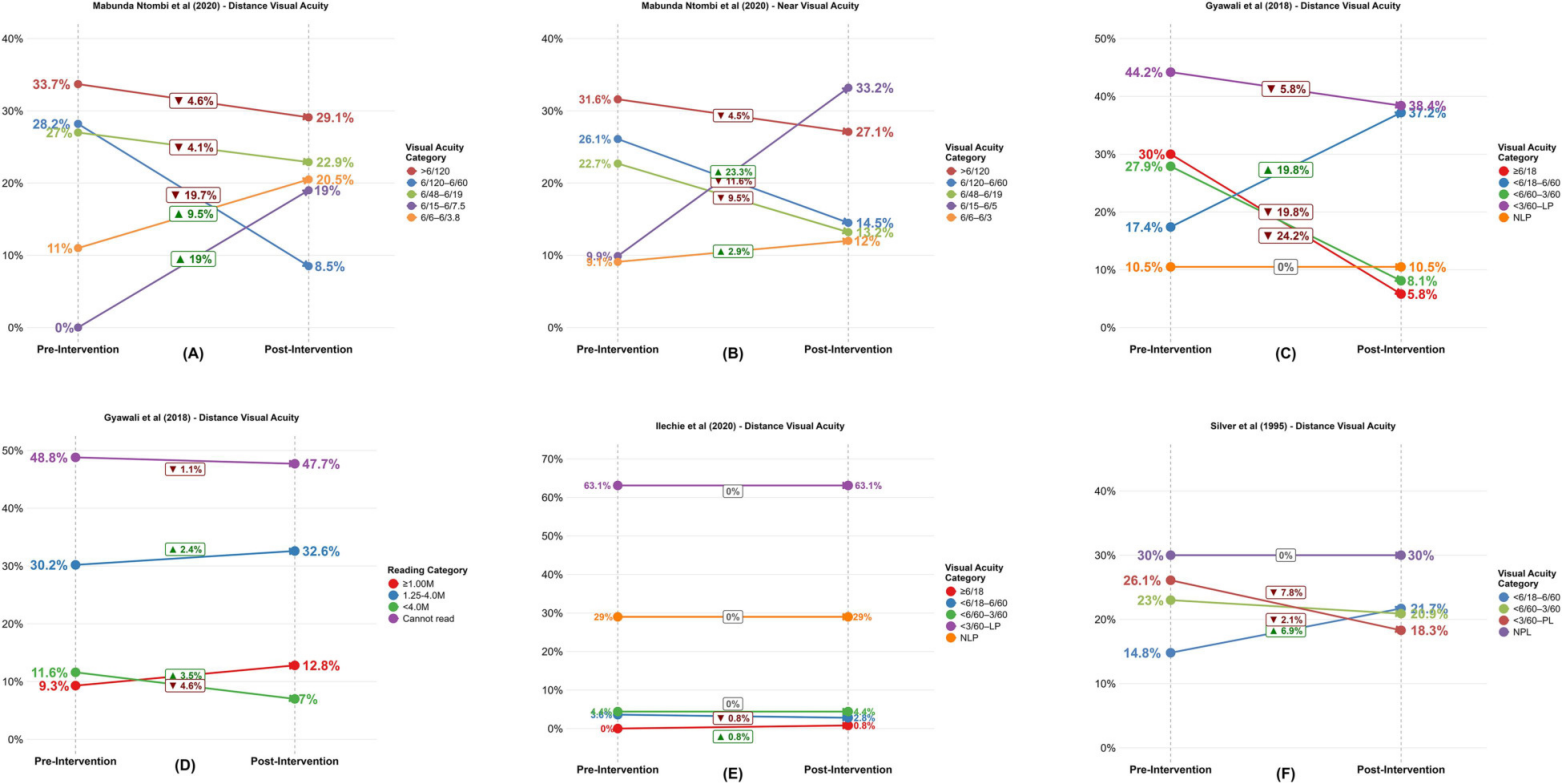
Pre- and post-intervention distance and near visual acuity distributions from 4 included studies. (A) Distance visual acuity for Mabunda Ntombi et al. (2020); (B) Near visual acuity for Mabunda Ntombi et al. (2020); (C) Distance visual acuity for Gyawali et al. (2018); (D) Near visual acuity for Gyawali et al. (2018); (E) Distance visual acuity for Ilechie et al. (2020); (F) Distance visual acuity for Silver et al. (1995).

In total, four studies reported on pre- and post-intervention visual acuity outcomes among individuals receiving low vision services across four SSA countries: South Africa, Eritrea, Ghana, and Kenya/Uganda (see Figure 3). Across the studies, visual acuity outcomes were assessed using clinical examinations, typically employing logMAR or Snellen charts, with results stratified into defined acuity categories for both distance and near vision.

Four studies reported both pre- and post-intervention data for distance visual acuity (21–23, 25), while two studies also presented data for near visual acuity (21, 23). The proportion of individuals achieving functional vision (≥6/18) improved significantly in some studies (21, 23). In South Africa, Mabunda et al. (21) reported an increase from 11.1% to 20.5% in the 6/6–6/3.8 category for distance vision. A similar trend was observed in Eritrea, where Gyawali et al. (23) reported an increase from 0% to 30.2% in the ≥6/18 category.

Improvements in near vision were also notable in these studies. Gyawali and colleagues (23) observed an increase from 9.3% to 22.1% in the ≥1.00 M category, while Mabunda et al. (21) reported a rise in the 6/6–6/3.8 category from 9.7% to 12.0%.

Across studies, reductions were observed in the proportion of individuals in poorer vision categories. For instance, Gyawali et al. (23) reported a decline in the <6/60–3/60 category from 27.9% to 2.3%, and Mabunda et al. (21) demonstrated a reduction in the >6/120 category from 33.7% to 29.1%. However, no significant changes were observed in the proportion of individuals with no light perception (NLP), which remained stable in studies by (22, 23, 25).

The overall magnitude of visual improvement varied between studies, with Mabunda and Gyawali showing the most substantial shifts. Conversely, Ilechie et al. (22) which assessed children in schools for the blind, reported minimal changes, with the proportion of individuals in the NLP and <3/60–LP categories remaining unchanged. Similarly, Silver et al. (25) found only modest gains, including a 6.9% increase in the <6/18–6/60 category and an 7.8% reduction in the <3/60–PL category (see Figure 3).

### 3.6 Barriers and facilitators to LVS provision and utilization

Barriers to the provision and utilization of LVS were consistently reported across all provider-based studies and in one patient-based study (see Table 5). At the provider level, key challenges included resource limitations such as the unavailability of low vision devices, lack of essential clinical equipment, and shortages of trained personnel. Financial constraints were also prominent, with low profitability of services and the high cost of devices acting as significant disincentives. Workflow-related challenges, including time-intensive care protocols and weak referral systems, further hindered service delivery. Additional barriers included knowledge gaps due to inadequate provider training and limited public awareness, as well as motivational issues, such as lack of interest or engagement among eye care practitioners.

**Table 5:** Barriers and facilitators to low vision service provision and utilization.

Category	References
Provider-level barriers	
Resource limitations Non-availability of low vision devices Lack of basic equipment Lack of personnel Lack of infrastructure Difficulty procuring equipment and devices	(13, 16, 17, 20)
Financial constraints Low profitability of LV care High cost of LV devices High cost of LV training	(13, 16, 17, 19)
Workflow challenges Time-consuming nature of LV care Lack of referral centers/patterns	(13, 16, 17, 19, 20)
Knowledge gaps Lack of provider training Lack of public awareness	(16, 17, 19)
Motivational factors Lack of interest/motivation Poor acceptance by patients	(16, 17, 19)
Patient-level barriers	
Financial High cost of devices Accessibility Geographic/distance barriers Unawareness of the presence of LV centers Psychosocial Difficulty adjusting to devices Social stigma Patient not seeing the need	(13, 16, 20)
Provider-level facilitators	
Training and education Advanced/specialized LV training Continuous professional development	(13, 16, 17)
Service Integration Central low vision centre Incorporating LV into eye clinics	(16, 17)
Awareness and affordability Public education campaigns Subsidized/affordable devices	(13, 16, 17)

From the patient perspective, reported barriers were primarily financial (e.g., the high cost of assistive devices), logistical (e.g., limited geographic access to service centers), and psychosocial (e.g., difficulty adapting to devices and the social stigma associated with low vision). These barriers reflect broader systemic issues in healthcare access but are compounded by disability-specific challenges, including marginalization and device dependency.

### 3.7 Risk of bias

Of the 11 studies, 10 studies were rated as having a low overall risk of bias, while one study (25) was judged to have moderate risk of bias due to unclear criteria for inclusion, which introduced uncertainty regarding the representativeness of the study sample and the reproducibility of the selection process. None of the studies were rated as high risk of bias. Confounding-related criteria (questions 5 and 6) were marked as “not applicable” for most studies, as these were primarily descriptive in nature and did not involve analytical methods requiring adjustment for confounding variables. These items were not weighted heavily in determining the overall risk of bias (see Supplementary File S2).

## 4 Discussion

This systematic review was conducted to address a critical gap in understanding the availability, distribution, access, utilization, and functional visual outcomes of LVS across SSA. Examined through this lens of the WHO Health Systems Framework (26), which identifies six interdependent building blocks including service delivery, health workforce, health information systems, medical products and technologies, financing, and leadership/governance as the essential architecture of any equitable and effective health system, the review's findings reveal consistent deficiencies across nearly all building blocks, collectively accounting for why LVS in SSA remain far below the level required to meet population need. These findings carry important implications not only for clinical practice but for health policy, given that the WHO World Report on Vision (2019) (27), identifies low vision as a leading cause of preventable disability in low- and middle-income countries, and have called for the integration of LVS into routine eye care as a prerequisite for achieving Universal Health Coverage (28). The review identified 11 eligible studies from both provider and patient perspectives, confirming a limited but growing evidence base across the region (13).

Provider-focused studies, conducted primarily in Ghana and Nigeria, reflected diverse clinical settings and professional backgrounds and demonstrated relatively high participation rates (Table 2). Patient-based studies were geographically broader—spanning South Africa, Ghana, Nigeria, Eritrea, Kenya, and Uganda—and were conducted across a range of service points, including hospitals, clinics, and schools for the blind. Ghana emerged as a regional leader in LVS provision (Table 3). Across the studies, optical aids were the most frequently reported service, with magnifiers being the most common. Non-optical aids, such as sunglasses and hats, were inconsistently available, and advanced technologies like closed-circuit televisions (CCTVs) were limited to a few centers. Four studies reported measurable improvements in visual acuity following low vision interventions, particularly for distance vision. However, access remained uneven, with patient populations facing varying degrees of service availability and quality (Table 4).

LVS in SSA are largely confined to foundational clinical assessments. While distance visual acuity (80%), near visual acuity (78.6%), and refraction (>68%) were commonly performed, these represent only the minimum threshold of a comprehensive low vision evaluation. The near-absence of contrast sensitivity testing, magnification needs assessment, and structured goal-setting is a notable clinical gap. The WHO low vision best-practice guidelines specify that effective low vision rehabilitation must extend well beyond acuity measurement to encompass functional vision assessment, patient-centred goal-setting, and psychosocial support (29). The gap between what is currently being delivered and what standards recommend reflects broader challenges in provider training, clinical tool availability, and the absence of standardized national protocols for LVS across most SSA countries. Patient history-taking was inconsistently performed, with limited documentation of disability-specific, psychosocial, or environmental factors. Needs assessments and goal-setting were similarly underutilized; only 20% of facilities assessed near-task needs, and 15.7% assessed mobility-related needs. Such omissions weaken the foundation for individualized rehabilitation planning and reduce the likelihood of meaningful functional improvements (30, 31).

The provision of low vision devices was highly variable and, in many settings, severely limited. In one South African clinic, telescopes and magnifiers were dispensed to 33.8% and 29.6% of patients, respectively (21), while many other facilities offered basic magnifying lenses or a narrow selection of aids (10). Non-optical aids were inconsistently available, with one study documenting 24.5% coverage (32), and several reporting none at all. This variability is not simply a supply problem—it reflects the absence of dedicated health financing mechanisms for assistive devices across the region. Without national procurement strategies, subsidised supply programmes, or inclusion of low vision aids in health insurance benefit packages, facilities are left to operate on an *ad hoc* basis that systematically disadvantages patients with fewer financial resources and those living in remote areas. Rehabilitation services were the least consistently delivered component of care. Monye and colleagues reported that 100% of patients received counseling on low vision device use, an essential step for promoting adoption and adherence (16). However, only 22% received structured training on device use. Vocational advice was provided to 89% of participants, and environmental modification training reached 67%, suggesting a relatively strong focus on functional support in this setting. Yet, mobility training was offered to only 22%, despite its importance in fostering independence (16).

In terms of functional outcomes, improvements in visual function were reported across four countries, with post-intervention visual acuity gains observed in both distance and near tasks. For example, in South Africa, the proportion of individuals achieving functional distance vision (≥6/18) increased from 11.1% to 39.5% after intervention (21). However, visual gains were less pronounced among children with profound visual impairment (21). In Kenya and Uganda, only modest improvements were recorded—7.4% in the <6/18–6/60 category and an 8.4% reduction in the <3/60–light perception group (25). These findings suggest that LVS are most effective when residual vision is present and underscore the importance of early detection and personalized rehabilitation goals (25). A critical limitation of the existing evidence base, however, is its near-exclusive reliance on visual acuity as the primary outcome. Visual acuity is an important clinical marker, but it does not capture whether patients are better able to read, work, study, or move independently following rehabilitation. Patient-reported outcomes, quality-of-life measures, and functional independence scales are largely absent from the SSA low vision service literature. This gap must be prioritized in future research if the true impact of these services is to be understood and communicated to policymakers.

Barriers to LVS provision and utilization were reported consistently across studies. At the provider level, challenges included limited availability of devices and basic equipment, high procurement costs, low service profitability, and insufficient human resources. Time-intensive service delivery, poor referral networks, and gaps in provider training further constrained implementation (17, 19). From the patient perspective, primary barriers included high device costs, long travel distances to service centers, and limited awareness of available services (25). Psychosocial factors, including stigma and difficulty adapting to assistive devices, were also cited, particularly in school settings. These challenges mirror broader healthcare access issues in SSA but are intensified by disability-related stigma and structural barriers (13, 16, 20).

Several facilitators were identified that offer a constructive starting point for that policy action. Integration of LVS into general eye care facilities emerged as one of the most pragmatic and scalable approaches, reducing the need for patients to travel to dedicated centers and embedding low vision care within existing clinical workflows. Public awareness campaigns, subsidized devices, and specialized provider training were reported to improve service reach and patient uptake (16, 17). Continuous professional development was especially noted as critical for expanding provider capacity and sustaining service delivery across the region (13, 16, 17). These facilitators suggest that LVS in SSA can be improved through deliberate policy commitment, coordinated investment in the existing workforce, and stronger integration into national eye health strategies and essential health packages.

### 4.1 Strengths and limitations

This review provides a comprehensive overview of low vision services in SSA by synthesizing evidence from both patient and provider perspectives. Strengths include a rigorous search strategy across multiple databases, inclusion of diverse study designs, and application of a validated quality assessment tool. However, several limitations should be noted. Most included studies were descriptive, with non-standardized outcome measures that limited comparability. Geographic coverage was uneven, with notable data gaps from several SSA countries, which may affect the generalizability of findings. Furthermore, functional outcome data were inconsistently reported, limiting the ability to assess the long-term impact of interventions as well as limited comparability and precluded statistical analysis, necessitating a descriptive approach to data presentation. In addition, whilst it is evident from this review that four studies reported on a total of 73 facilities providing low vision services, this estimate should be interpreted with caution. The included studies from Ghana did not consistently report the names or identifiers of the facilities assessed, making it difficult to determine whether there was overlap between studies. As a result, it is possible that some facilities were counted more than once, which may overestimate the true number and geographic distribution of low vision service centers. Although Ghana appears to have the highest representation of facilities, this finding may partly reflect duplication in reporting rather than a fully distinct network of services.

## 5 Conclusion

This review reveals substantial gaps in the availability, accessibility, and utilization of low vision services across SSA. Although optical aids and basic clinical assessments are increasingly offered, specialized evaluations, rehabilitation training, and comprehensive patient-centered care remain limited. Barriers—ranging from infrastructural constraints and financial barriers to provider training deficits and social stigma—continue to hinder service delivery. Nonetheless, the documented improvements in visual function underscore the potential of LVS when properly implemented. Strengthening the integration of LVS into routine eye care, expanding provider training, and adopting inclusive public health strategies are essential to improving functional outcomes and ensuring equitable access to low vision care across the region.

## Patient and public involvement

Patients and members of the public were not directly involved in the design, conduct, or reporting of this systematic review, as it was based exclusively on the analysis of previously published data. However, the research question was shaped by service gaps in low vision care identified in existing literature from SSA.

## Data Availability

The original contributions presented in the study are included in the article/Supplementary Material, further inquiries can be directed to the corresponding authors.

## Supplementary material

Supplementary File S1 Search terms.

Supplementary File S2 Risk of bias.

Supplementary File S3 Excluded studies.

## References

[1] Bourne R Steinmetz JD Flaxman S Briant PS Taylor HR Resnikoff S. Trends in prevalence of blindness and distance and near vision impairment over 30 years: an analysis for the global burden of disease study. Lancet Glob Health. (2021) 9(2):e130–43. 10.1016/S2214-109X(20)30425-3 33275950 PMC7820390

[2] Vision Loss Expert Group of the Global Burden of Disease Study, The GBD Blindness and Vision Impairment Collaborators. Prevalence of blindness and visual impairment in sub-Saharan Africa in 2020: magnitude and temporal trends. Systematic review and meta-analysis. Ophthalmic Epidemiol. (2026) 33(1):43–53. 10.1080/09286586.2025.2474654 40127261

[3] Markowitz M. Occupational therapy interventions in low vision rehabilitation. Can J Ophthalmol. (2006) 41(3):340–7. 10.1139/I06-020 16767190

[4] Massof RW. A model of the prevalence and incidence of low vision and blindness among adults in the US. Optom Vis Sci. (2002) 79(1):31–8. 10.1002/j.1538-9235.2002.tb01405.x 11828896

[5] Stelmack J. Emergence of a rehabilitation medicine model for low vision service delivery, policy, and funding. Optometry. (2005) 76(5):318–26. 10.1016/S1529-1839(05)70315-8 15884422

[6] Stelmack JA Stelmack TR Massof RW. Measuring low-vision rehabilitation outcomes with the NEI VFQ-25. Invest Ophthalmol Visual Sci. (2002) 43(9):2859–68. 12202503

[7] Chiang PPC O’Connor PM Keeffe JE. Low vision service provision: a global perspective. Expert Rev Ophthalmol. (2007) 2(5):861–74. 10.1586/17469899.2.5.861

[8] Andrade Selibe R. An exploration of strategies for an uncertain future for CNIB given aging with vision loss (2018).

[9] Vishal B Kr BG Sourav K Animesh M. Barriers in attaining low-vision care services: a narrative review. Delta J Ophthalmol. (2022) 23(4):221–5. 10.4103/djo.djo_15_22

[10] Xulu-Kasaba ZN Kalinda C. Prevalence of blindness and its major causes in sub-Saharan Africa in 2020: a systematic review and meta-analysis. Br J Vis Impair. (2022) 40(3):563–77. 10.1177/02646196211055924

[11] Naidoo K Kempen JH Gichuhi S Braithwaite T Casson RJ Cicinelli MV. Prevalence and causes of vision loss in sub-Saharan Africa in 2015: magnitude, temporal trends and projections. Br J Ophthalmol. (2020) 104(12):1658–68. 10.1136/bjophthalmol-2019-315217 32229517

[12] Abraham CH van Staden D Rampersad N. Barriers and enablers to low vision care and rehabilitation in sub-Saharan Africa within a global context. Clin Exp Optom. (2024) 107(1):3–13. 10.1080/08164622.2023.2254766 37993138

[13] Akuffo KO Osei Duah Junior I Acquah EA Abadua Mensah E Andoh AKA Kumah DB. Low vision practice and service provision among optometrists in Ghana: a nationwide survey. Ophthalmic Epidemiol. (2025) 32(1):1–8. 10.1080/09286586.2024.2317816 38451021

[14] Moher D Liberati A Tetzlaff J Altman DG, PRISMA Group. Preferred reporting items for systematic reviews and meta-analyses: the PRISMA statement. Int J Surg. (2010) 8(5):336–41. 10.1016/j.ijsu.2010.02.007 20171303

[15] Barker TH Hasanoff S Aromataris E Stone JC Leonardi-Bee J Sears K. The revised JBI critical appraisal tool for the assessment of risk of bias for analytical cross-sectional studies. JBI Evid Synth. (2026) 24(3):401–8. 10.11124/JBIES-24-00523 40521701

[16] Monye H Kyari F Momoh R. A situational report on low vision services in tertiary hospitals in south-east Nigeria. Niger J Clin Pract. (2020) 23(7):919–27. 10.4103/njcp.njcp_375_19 32620720

[17] Nyankerh C Agyekum S Boateng A Appah M. Low vision service delivery by optometrists in Ghana. Priv Pact. (2019) 101:78–9.

[18] Boadi-Kusi SB Ntodie M Mashige KP Owusu-Ansah A Antwi Osei K. A cross-sectional survey of optometrists and optometric practices in Ghana. Clin Exp Optom. (2015) 98(5):473–7. 10.1111/cxo.12291 25944332

[19] Okoye OI Aghaji A Umeh R Nwagbo D Chuku A. Barriers to the provision of clinical low-vision services among ophthalmologists in Nigeria. Vis Impair Res. (2007) 9(1):11–7. 10.1080/13882350701198702

[20] Kyeremeh S Mashige KP. Availability of low vision services and barriers to their provision and uptake in Ghana: practitioners’ perspectives. Afr Health Sci. (2021) 21(2):896–903. 10.4314/ahs.v21i2.51 34795749 PMC8568257

[21] Mabunda NA Parsad A Mashige KP Xulu-Kasaba ZN Mthembu MG Mazibuko NS. A profile of patients presenting at a low vision clinic in a resource-limited setting. Afr Vis Eye Health. (2020) 79(1):1–7. https://hdl.handle.net/10520/EJC-1d77dd74bc

[22] Ilechie A Wanye S Abraham CH Sarpong JB Abu E Abokyi S. Inter-regional trends in causes of childhood blindness and low vision in Ghana. Clin Exp Optom. (2020) 103(5):684–92. 10.1111/cxo.13041 31916287

[23] Gyawali R Moodley VR. Need for optical intervention in children attending a school for the blind in Eritrea. Clin Exp Optom. (2018) 101(4):565–70. 10.1111/cxo.12601 28952171

[24] Eze C Okoye O Okoye O Nwachukwu N Okoloagu N Onwasigwe E. Profile of low vision patients in a resource-poor underserved setting of a developing country. Open J Ophthalmol. (2018) 8(02):120–31. 10.4236/ojoph.2018.82016

[25] Silver J Gilbert CE Spoerer P Foster A. Low vision in east African blind school students: need for optical low vision services. Br J Ophthalmol. (1995) 79(9):814–20. 10.1136/bjo.79.9.814 7488599 PMC505266

[26] World Health Organization. The WHO health systems framework (2017). Available online at: https://www.who.int/publications/b/31426 (Accessed April 20, 2026)

[27] World Health Organization. World report on vision. Geneva: WHO (2019). Available online at: https://wkc.who.int/resources/publications/i/item/world-report-on-vision (Accessed April 20, 2026)

[28] World Health Organization. Universal health coverage (2025). Available online at: https://www.who.int/news-room/fact-sheets/detail/universal-health-coverage-(uhc) (Accessed April 20, 2026)

[29] World Health Organization. Package of eye care interventions (2022). Available online at: https://www.who.int/publications/i/item/9789240048959 (Accessed April 20, 2026)

[30] Şahlı E İdil A. A common approach to low vision: examination and rehabilitation of the patient with low vision. Turk J Ophthalmol. (2019) 49(2):89–98. 10.4274/tjo.galenos.2018.65928 31055894 PMC6517854

[31] Luu W Kalloniatis M Bartley E Tu M Dillon L Zangerl B. A holistic model of low vision care for improving vision-related quality of life. Clin Exp Optom. (2020) 103(6):733–41. 10.1111/cxo.13054 32128871

[32] Xulu-Kasaba Z Mashige K Naidoo K. Knowledge, attitudes and practices of eye health among public sector eye health workers in South Africa. Int J Environ Res Public Health. (2021) 18(23):12513. 10.3390/ijerph182312513 34886238 PMC8656467

