# Supplementary material for: Low vision services in sub-Saharan Africa: a systematic review
Source: Front Health Serv. 2026 Jun 3;6:1839924. doi: 10.3389/frhs.2026.1839924 (PMC13272409; doi:10.3389/frhs.2026.1839924)
Supplement: Supplementary File S2 — Risk of bias. [file Supplementaryfile2.docx]

| **Concept** | **Search terms** |
| --- | --- |
| Low vision | low vision OR visual impairment OR vision loss OR visual disability OR vision impairment OR subnormal vision OR diminished vision |
| Low vision services | rehabilitation OR low vision services OR vision rehabilitation OR low vision aid* OR low vision rehabilitation OR special education OR inclusive education OR barrier* OR limitations OR intervention* OR optical aid* OR non-optical aid* |
| Sub-Saharan Africa | "Africa South of the Sahara" OR "sub-Saharan Africa" OR Angola OR Benin OR Botswana OR Burkina Faso OR Burundi OR "Cabo Verde" OR Cameroon OR "Central African Republic" OR Chad OR Comoros OR Congo OR "Democratic Republic of Congo" OR Djibouti OR "Equatorial Guinea" OR Eritrea OR Eswatini OR Ethiopia OR Gabon OR Gambia OR Ghana OR Guinea OR "Guinea-Bissau" OR Ivory Coast OR Kenya OR Lesotho OR Liberia OR Madagascar OR Malawi OR Mali OR Mauritania OR Mozambique OR Namibia OR Niger OR Nigeria OR Rwanda OR "Sao Tome and Principe" OR Senegal OR Seychelles OR "Sierra Leone" OR Somalia OR "South Africa" OR "South Sudan" OR Sudan OR Tanzania OR Togo OR Uganda OR Zambia OR Zimbabwe |
